# Ureteral metastasis from locally invasive, castrate-resistant prostate cancer: A Case report

**DOI:** 10.1016/j.eucr.2021.101780

**Published:** 2021-07-13

**Authors:** Zachary C. Janatpour, Santosh Shanmuga, Edwin Gandia, Inger L. Rosner

**Affiliations:** a F. Edward Hébert School of Medicine; Uniformed Services University of the Health Sciences, 4301 Jones Bridge Rd, Bethesda, MD 20814, United States; b Division of Urology, Department of Surgery, Uniformed Services University of the Health Sciences, Walter Reed National Military Medical Center, 8901 Rockville Pike, Bethesda, MD 20889, United States; c Department of Pathology, Uniformed Services University of the Health Sciences, Walter Reed National Military Medical Center, 8901 Rockville Pike, Bethesda, MD 20889, United States; d Department of Urology, Inova Fairfax Hospital, 8081 Innovation Park Dr, Fairfax, VA 22031, USA

**Keywords:** Prostate cancer, Ureteral metastasis, Metastatic prostate cancer, Castrate-resistant prostate cancer, Nephroureterectomy

## Abstract

Within the text we describe a 66-year-old male with a history of prostate cancer (PCa), incidentally found to have left-sided hydronephrosis and a left ureteral lesion. Ureteroscopy was employed to visualize the lesion, a biopsy was taken, and a double J stent was placed. The lesion was of prostatic origin and the patient was subsequently started on androgen deprivation therapy (ADT). 6 months following the procedure, the patient's PSA had decreased and his hydronephrosis had resolved. We are the first to report treating a ureteral metastasis from PCa and its associated hydronephrosis solely with ADT and double J stenting, respectively.

## Introduction

As the incidence of metastasis to the ureters remains exceedingly rare, disease management for patients with ureteral metastases is often guided by the few published experiences of others. Thus, it is imperative that additional cases be documented in a public domain. The following Case describes an incidence of ureteral metastasis from locally invasive, castrate-resistant prostate cancer that was managed without resection.

## Case presentation

The patient is a 66-year-old male who presented to clinic for an elevated PSA (3.79 ng/mL in 2014 to 14.95 ng/mL in 2015). Transrectal ultrasound guided prostate biopsy revealed high volume, high-risk disease with 11 of 12 cores positive and a Gleason score of 5 + 5 = 10. He underwent robot-assisted radical prostatectomy that was complicated by a 4 mm rectal laceration. Final pathological staging was pT4N0R1 with positive margins and rectal invasion. The patient was subsequently started on androgen deprivation therapy (ADT) with leuprolide acetate administered every 3 months, and his PSA dropped to a nadir of 1.33mg/mL.

Surveillance MRI in 2016 revealed a T2-intense nodular region at the posterior inferior bladder. Additionally, PET scan revealed radiotracer accumulation within a single vertebral body. However, concerns for bony malignancy were low at that time due to the solitary nature of the finding. In late 2017 the patient developed castrate resistant disease due to increasing PSA with a PSA doubling time of 5.6 months. MRI performed in 2018 revealed enlargement of the previously mentioned T2-intense nodular region at the posterior inferior bladder. In late 2018 the patient was referred for enrollment in the NIH Clinical protocol NCT 03315871 for treatment of combination immunotherapy involving vaccines (Prostvac and CV301) and MSB0011359C [an anti-PD-L1 antibody (avelumab) with TGF beta-Trap molecule].

**Fig. 1:**
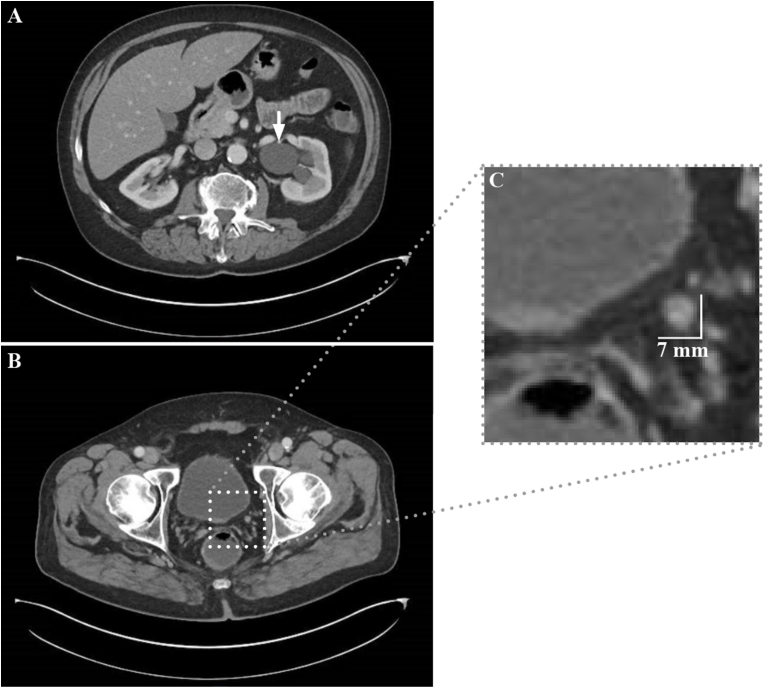
Computed tomography (CT) of the abdomen and pelvis. Cross-sectional imaging using computed tomography (CT) of the abdomen and pelvis with IV contrast shows marked left hydroureteronephrosis and an enhancing ureteral mass in the distal left ureter.

In early 2020, a computerized tomography (CT) revealed development of left hydroureteronephrosis (Fig. 1) that traced to the level of an enhancing lesion in the left distal ureter, as well as the presence of enlarging sclerotic vertebral lesions; both concerning for metastasis. The patient was started on Apalutamide in addition to depot goserelin, and scheduled for diagnostic ureteroscopy.

**Fig. 2:**
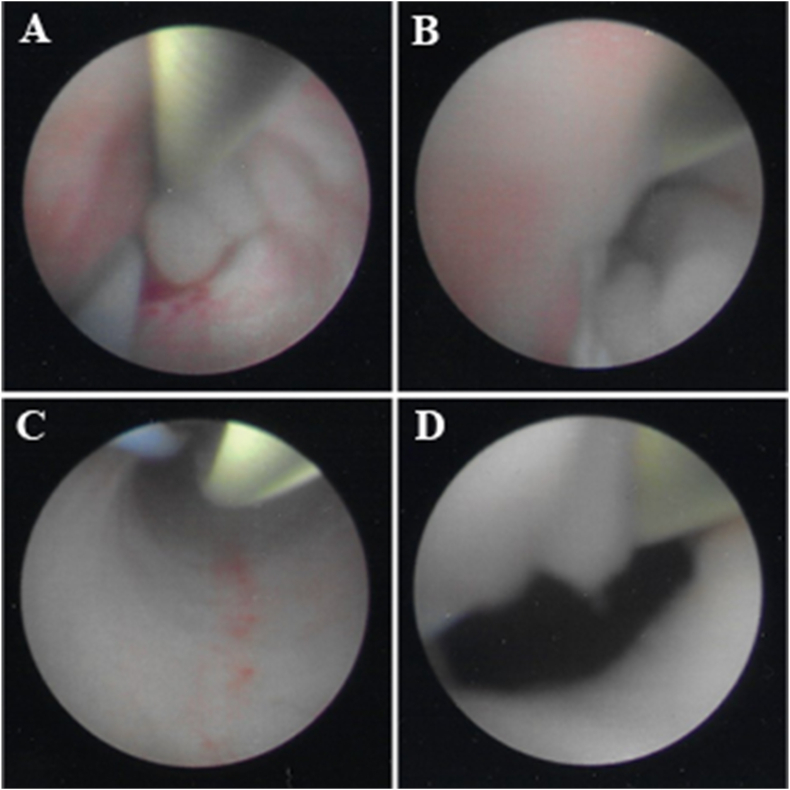
Diagnostic ureteroscopy. Intra-operative images from the left diagnostic ureteroscopy show a bulbous ureteral mass (A, B), with normal, dilated ureter proximal to the mass (C). A post-biopsy image of the mass (D) is also shown.

Cystoscopy was notable for post-prostatectomy changes and a bladder neck mass consistent with his recurrent prostate cancer. Retrograde pyelogram of the left ureter revealed marked hydroureteronephrosis and a torturous ureter. Left ureteroscopy revealed a bulbous mass in the left distal ureter (Fig. 2A and B), with normal ureter observed distally and dilated ureter observed proximally (Fig. 2C and D). Biopsies were taken using ureteral cold cup biopsy forceps. A left ureteral 6Fr x 28cm double J stent was placed successfully.

**Fig. 3:**
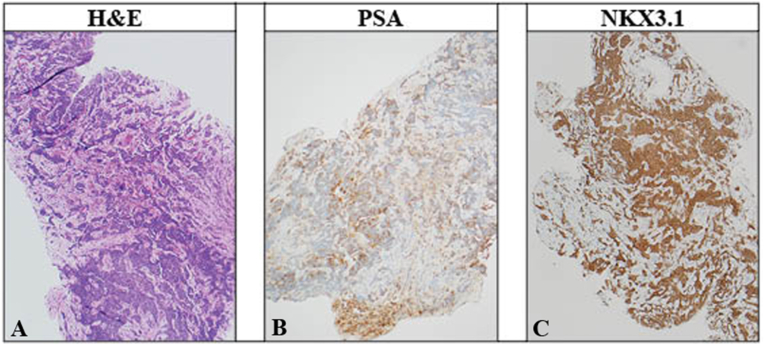
Histopathologic analysis suggest prostatic origin of ureteral biopsy. A) Hematoxylin and Eosin (H&E) stain at of the biopsy material showing a prominent cell infiltrate with severe cautery artifact. B) Prostate specific antigen (PSA) immunohistochemical stain of ureteral biopsy. Note the patchy, moderate to strong immunoreactivity in the cells of interest. C) NKX3.1 immunohistochemical stain. The cells show strong, diffuse immunoreactivity. PSA and NKX3.1 immunoreactivity favors metastatic carcinoma of prostatic origin.

This biopsy specimen consisted of multiple small tissue fragments characterized by the presence of a cell infiltrate with severe crush artifact. Evaluation of morphology and architecture was precluded by the presence of this severe crush artifact (Fig. 3A). An immunohistochemical panel of PSA, NKX3.1, CD45, SOX-10, synaptophysin and chromogranin with appropriately reactive controls was performed on the submitted biopsy material. CD45 highlighted the background inflammatory cells. PSA showed mild to moderate immunoreactivity, while NKX3.1 showed strong immunoreactivity in the cells of interest (Fig. 3B and C). Chromogranin, synaptophysin and SOX-10 were negative. The immunohistochemical profile was consistent with the diagnosis of metastatic carcinoma of prostatic origin.

6 months following ADT and double J stenting, the patient remained asymptomatic, the patient's PSA showed a substantial decrease, and repeat CT showed no hydroureteronephrosis.

## Discussion

Occurrence of ureteral metastasis from prostate cancer remains exceedingly rare, with approximately 50 cases documented over the last century. Gandaglia et al. assessed distribution of metastasis in a cohort of 74,826 patients with metastatic PCa and described the most common metastatic sites as bone (84%), distant lymph nodes (10.6%), liver (10.2%), and thorax (9.1%).^1^ For PCa patients with bone metastases, the most common sites of secondary metastases were liver (39.1%), thorax (35.2%), distant lymph nodes (24.6%), and brain (12.4%).^1^ In fact, the most common source of ureteral metastasis are breast cancer, gastric cancer, and colorectal cancer; rather than PCa.^2^ Due to the infrequent occurrence of ureteral metastasis from PCa, a paucity of literature exists with regard to understanding risk factors and pathophysiology that contribute to acquiring ureteral metastasis.

Among patients presenting with a ureteral mass, it is common for surgeons to perform nephroureterectomy (NU) rather than diagnostic ureteroscopy with biopsy. Ureteroscopy is often difficult in patients with ureteral strictures, and nephroureterectomy allows for both reduction of disease burden and biopsy to occur within one step. However, as in our Case, we were able to easily access the ureter via ureteroscopy which allowed for visualization both proximal and distal to the lesion, and easy acquisition of a biopsy. Similar to case reports by Gupal et al., in 2009, Schneider et al., in 2012, and Munshi et al., in 2019, we decided against nephroureterectomy due to concerns of post-surgical complications in a patient with extensive extra-prostatic disease burden.^3^, ^4^, ^5^ Unlike any of the aforementioned cases, we placed 6Fr x 28cm double J stent to relieve the patient's hydronephrosis, rather than performing a nephrostomy, and did not resect the lesion.

## Conclusion

As best we can tell, we are the first to report treating a ureteral metastasis from PCa and its associated hydronephrosis solely with ADT and double J stenting, respectively.

## Funding

This research did not receive any specific grant from funding agencies in the public, commercial, or not-for-profit sectors.

## DoD disclaimer

The opinions and assertions contained herein are the private opinions of the authors and are not to be construed as reflecting the views of the Uniformed Services University of the Health Sciences, the U.S. Department of Defense, or the U.S. Department of the Army.

## Author contribution

All authors have made substantial contributions to the acquisition of data, the interpretation of data, and both drafting and revising the article critically for important intellectual content. All authors have granted approval of the final version to be submitted.

## Declaration of competing interest

None.
